# Supplementary figures and images for: Polyphenolic extract from Punica granatum peel causes cytoskeleton-related damage on Giardia lamblia trophozoites in vitro
Source: PeerJ. 2022 Apr 27;10:e13350. doi: 10.7717/peerj.13350 (PMC9055998; doi:10.7717/peerj.13350)

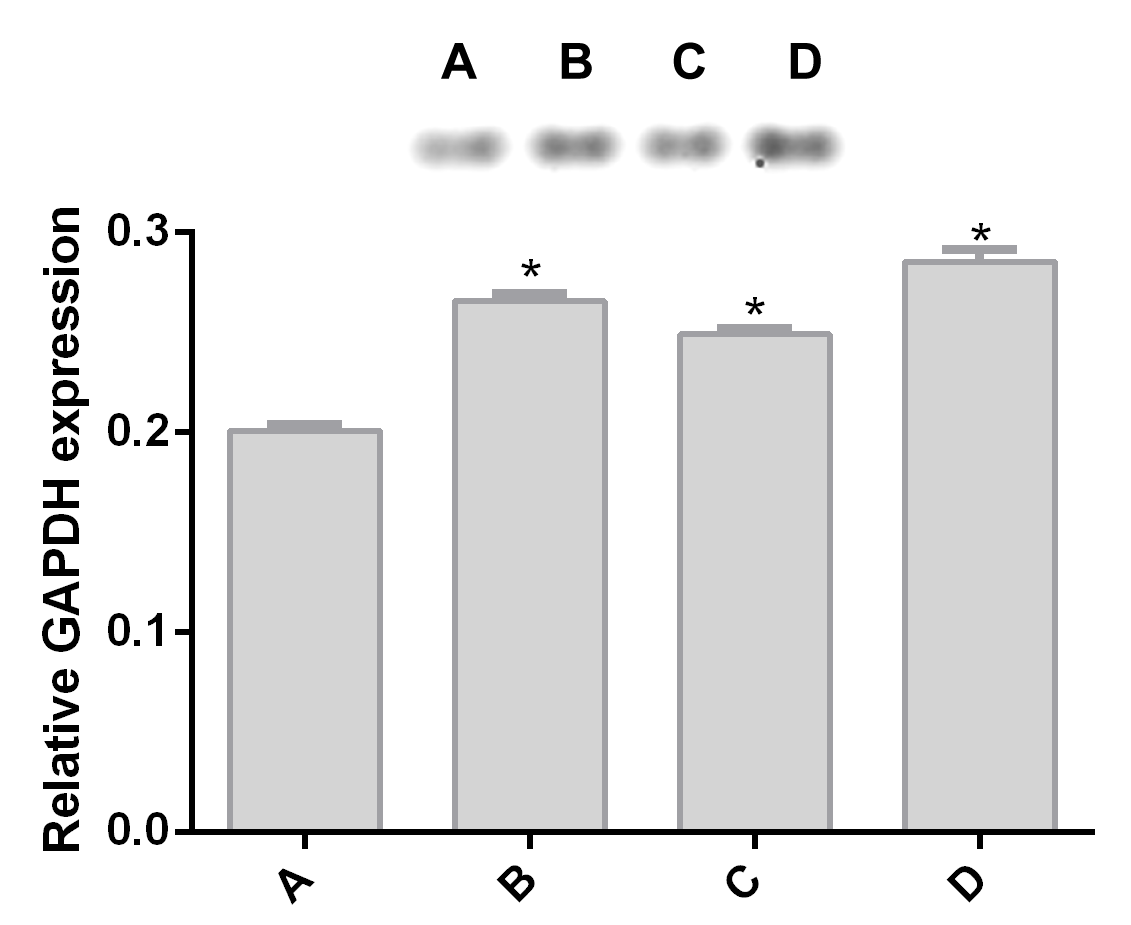

Supplement: Supplemental Information 2 — Image of western blotting (shown above) and densitometric analysis of (A) DMSO, (B) 150 µg/mL, (C) 175 µg/mL, and (D) 200 µg/mL treated cells. Data analyzed with GraphPad 6 software, *p ≤ 0.0001 [file peerj-10-13350-s002.png]
